# Supplementary material for: NEDD4 and NEDD4L regulate Wnt signalling and intestinal stem cell priming by degrading LGR5 receptor
Source: EMBO J. 2019 Dec 23;39(3):e102771. doi: 10.15252/embj.2019102771 (PMC6996568; doi:10.15252/embj.2019102771)
Supplement: Supplementary file 1 — Appendix [file EMBJ-39-e102771-s001.pdf]

Appendix - Table of contents

Appendix Fig. S1

Appendix Fig. S2

Appendix Fig. S1

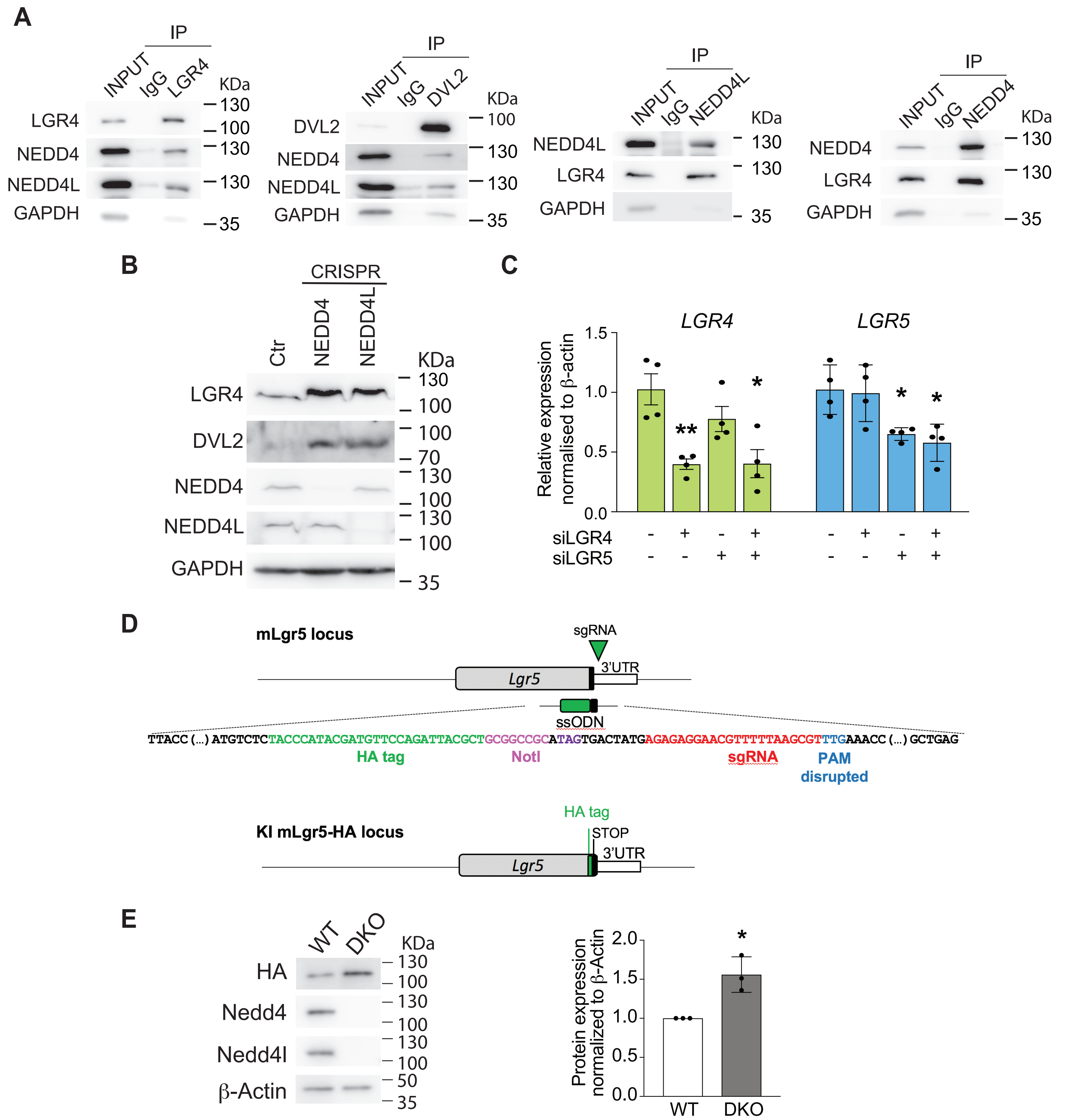

Appendix Fig. S1. NEDD4 and NEDD4L degrade LGR4, LGR5 and DVL2 at the endogenous level.
(A) HEK293T were immunoprecipitated with LGR4, DVL2, NEDD4 or NEDD4L antibodies followed by Western blot analysis using the indicated antibodies.
(B) Cell lysates of HEK293T control (Ctr) and the indicated CRISPR clones were analysed by western blotting using the indicated antibodies.
(C) mRNA expression of LGR4 and LGR5 genes was analysed by qRT-PCR HEK293T cells transfected with the indicated siRNAs. Data are presented as fold change normalized to -actin control in triplicate (n=3 per condition). Error bars represent ± standard error. P-values were determined using the unpaired two-sided t-test. (*p<0.05; **p<0.01).
(D) Schematic representation of the mouse Lgr5-HA tag knockin strategy.
(E) Lysates from WT and DKO organoids targeted with Lgr5-HA tag knockin were analysed by western blotting using the indicated antibodies. The graph represents the quantitation of the HA protein expression in WT and DKO targeted organoids normalized to -actin control performed in triplicate. Error bars represent ± standard error. P-values were determined using the unpaired two-sided t-test. (*p<0.05).

Appendix Fig. S2

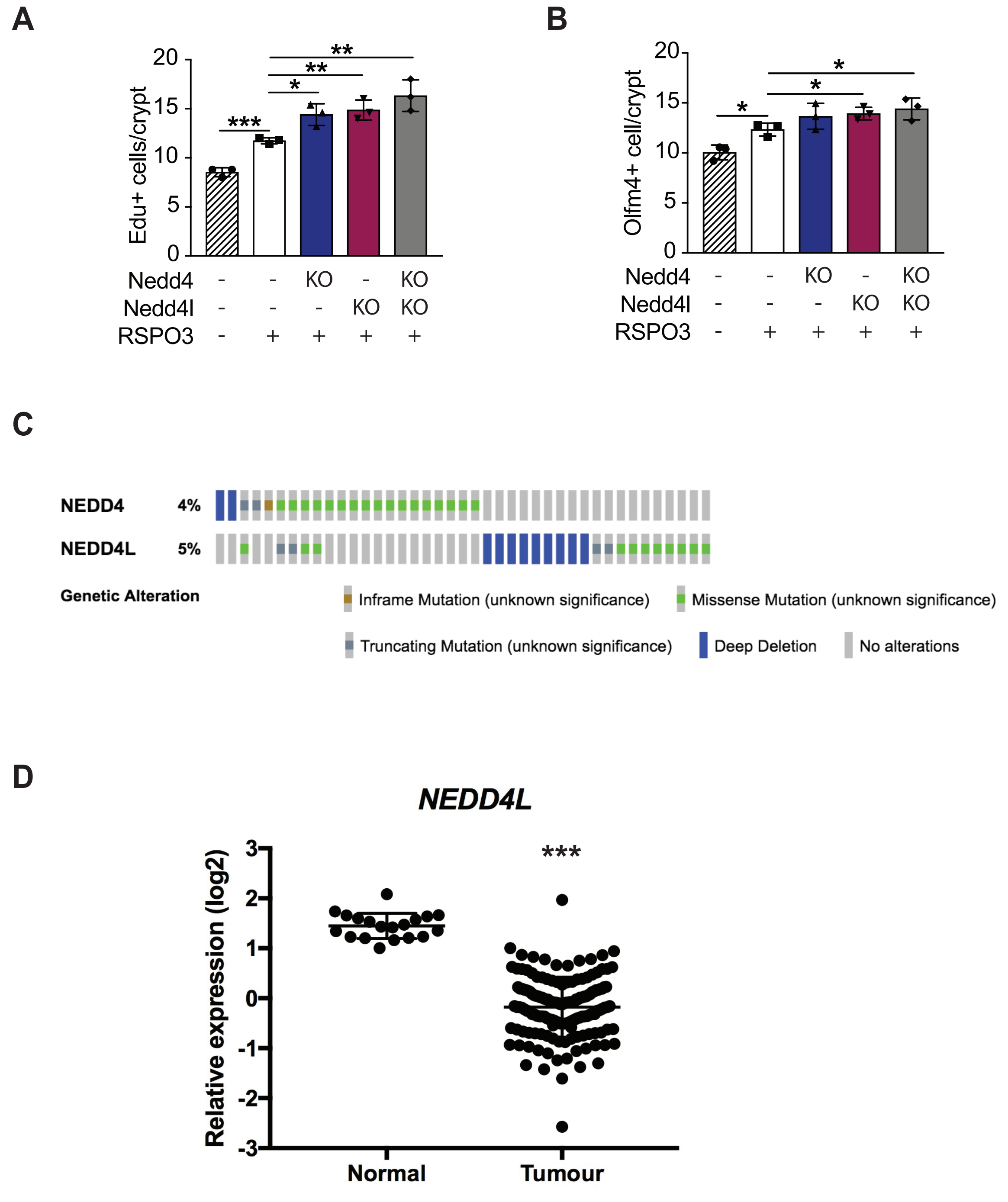

Appendix Fig. S2. Loss of Nedd4 and Nedd4l increases crypt proliferation and ISC expansion upon RSPO stimulation.

(A-B) Quantitation of Edu+ (A) and Olfm4+ (B) cells in the intestinal crypts from the indicated mice. Each dot represents the average of at least 20 crypts per animal. Data are mean ± standard error. n=3 per group. P-values were determined using the unpaired two-sided t-test. (*p<0.05; **p<0.01; ***p<0.001).

(C) Genetic alteration of NEDD4 and NEDD4L in human colorectal cancer (CRC) patients (source from cBioPortal). Each patient sample is represented by a bar and each colour indicated specific genetic alteration as indicated. Only patients with alterations are shown. The frequency of gene alteration is represented as a percentage.

(D) Relative mRNA expression of NEDD4L between 19 normal and 154 CRC patients (data obtained from the Cancer Genome Atlas) (Cancer Genome Atlas, 2012). P-values were determined using the unpaired two-sided t-test. (***p<0.001).
