# Supplementary material for: NEDD4 and NEDD4L regulate Wnt signalling and intestinal stem cell priming by degrading LGR5 receptor
Source: EMBO J. 2019 Dec 23;39(3):e102771. doi: 10.15252/embj.2019102771 (PMC6996568; doi:10.15252/embj.2019102771)
Supplement: Supplementary file 2 — Expanded View Figures PDF [file EMBJ-39-e102771-s002.pdf]

Expanded View Figures

Figure EV1. Short-term deletion of Nedd4 and/or Nedd4l in the intestine.

A Schematic representation of the crypt–villus axis of the small intestine. mRNA expression of the indicated genes was analysed by qRT–PCR in villi and crypts isolated from wild-type intestine. Data are presented as fold change normalised to *Hprt1* control (n = 4 per group). Error bars represent ± standard error.

B Representative images of WT, Nedd4 cKO, Nedd4l cKO and DKO proximal intestine collected at 50 dpi stained for H&E, PAS, Cyclin D1, Sox9 and Edu (n = 3 per group). Scale bars, 50 μm.

C mRNA expression of the indicated genes was analysed by qRT–PCR in crypts from small intestinal organoids isolated from the correspondent WT and DKO mice. Data are presented as fold change normalised to Hprt1 control in triplicate (n = 3 per condition). Error bars represent ± standard error.

Data information: P-values were determined using the unpaired two-sided t-test (*P < 0.05; **P < 0.01; ***P < 0.001).

▶

 © 2019 The Authors

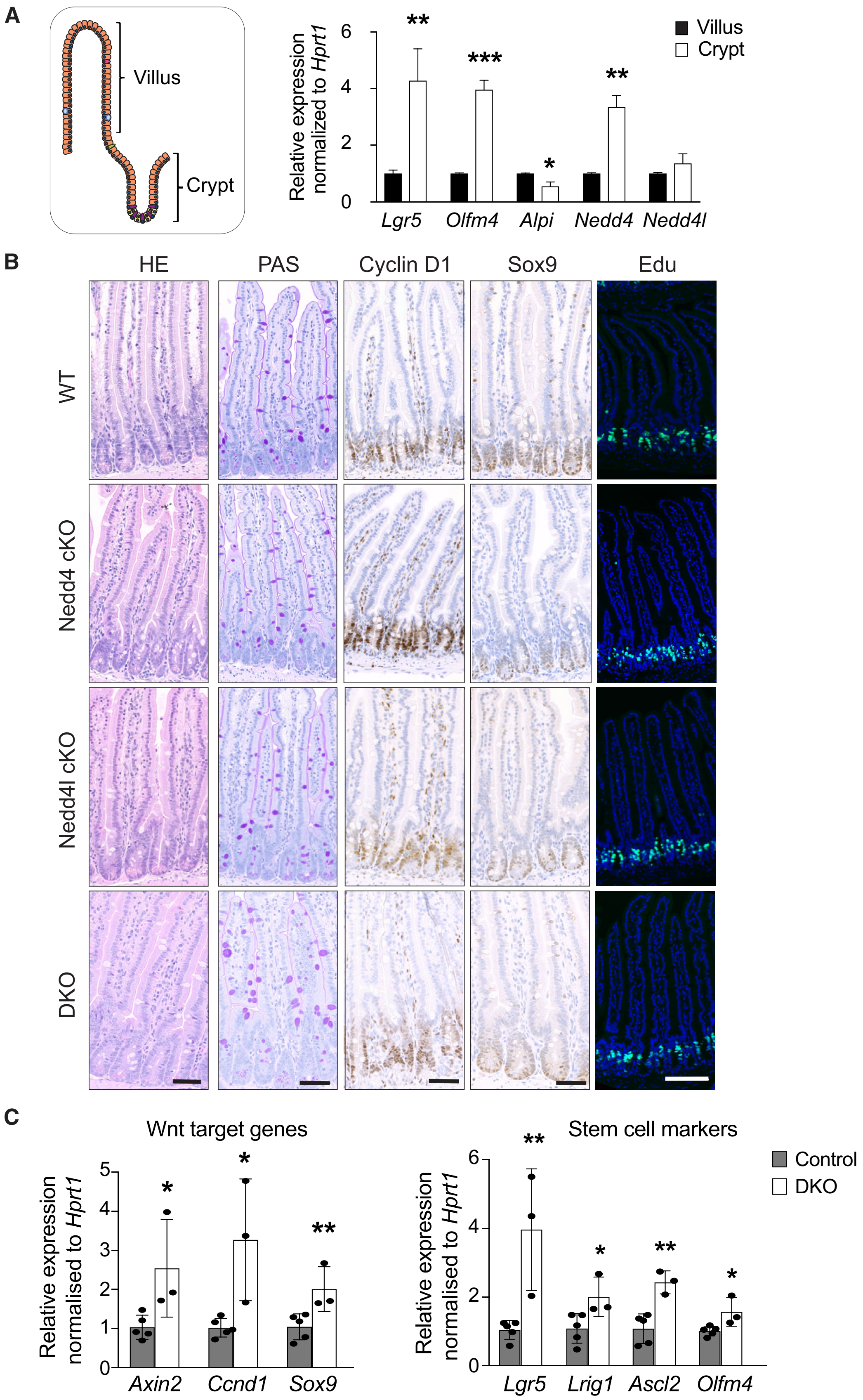
A
Villus
Crypt
Relative expression normalized to Hprt1
6
4
2
0
**
***
*
**
Villus
Crypt
Lgr5
Olfm4
Alpi
Nedd4
Nedd4l
B
HE
PAS
Cyclin D1
Sox9
Edu
WT
Nedd4 cKO
Nedd4l cKO
DKO
C
Wnt target genes
Relative expression normalised to Hprt1
5
4
3
2
1
0
*
*
**
Axin2
Ccnd1
Sox9
Stem cell markers
6
4
2
0
**
*
**
*
Lgr5
Lrig1
Ascl2
Olfm4
Control
DKO

Figure EV1.

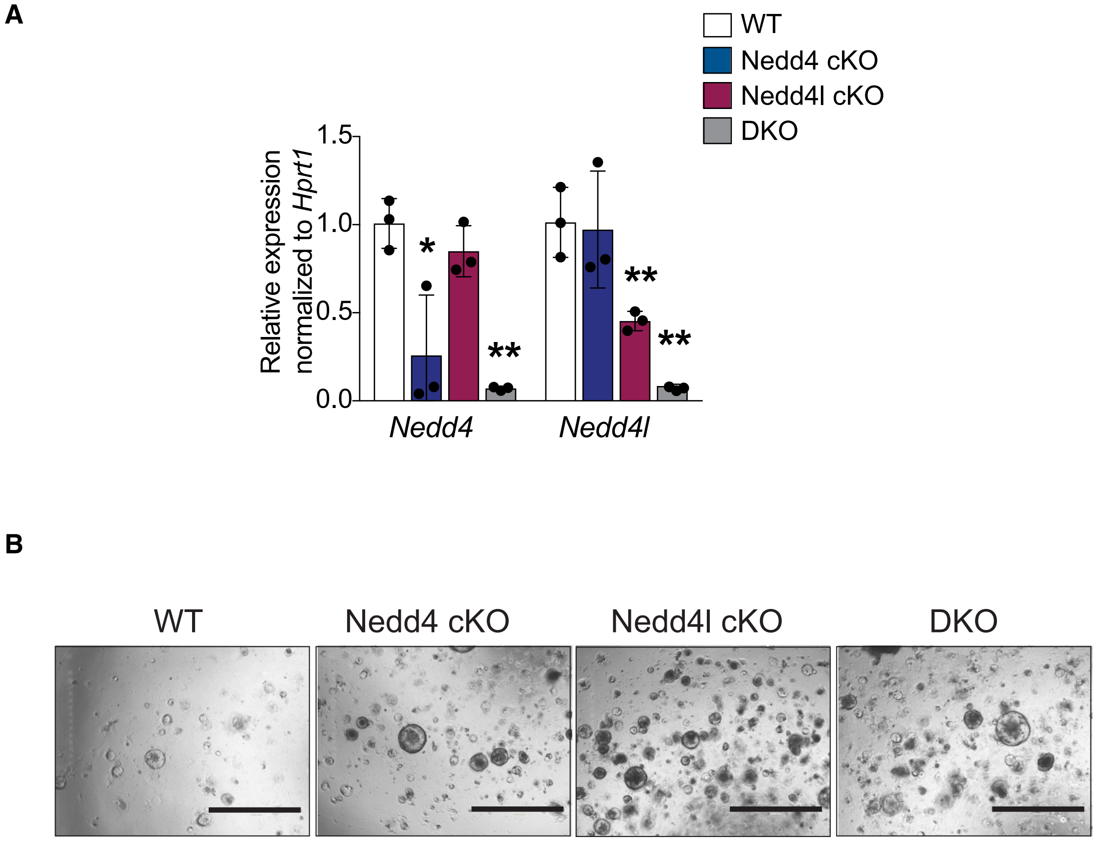

Figure EV2. Characterisation of *in vitro* phenotype using small intestinal organoids.

A mRNA expression of the indicated genes was analysed by qRT–PCR in small intestinal organoids isolated from the correspondent WT, Nedd4 cKO, Nedd4l cKO and DKO mice. Data are presented as fold change normalised to Hprt1 control in triplicate ($n = 3$ per condition). Error bars represent ± standard error. *P*-values were determined using the unpaired two-sided *t*-test (*$P < 0.05$; **$P < 0.01$).

B Representative photographs of colony formation assay of organoids derived from WT, Nedd4 cKO, Nedd4l cKO and DKO intestine at 5 dpi. Scale bar, 1,000 µm.

 © 2019 The Authors

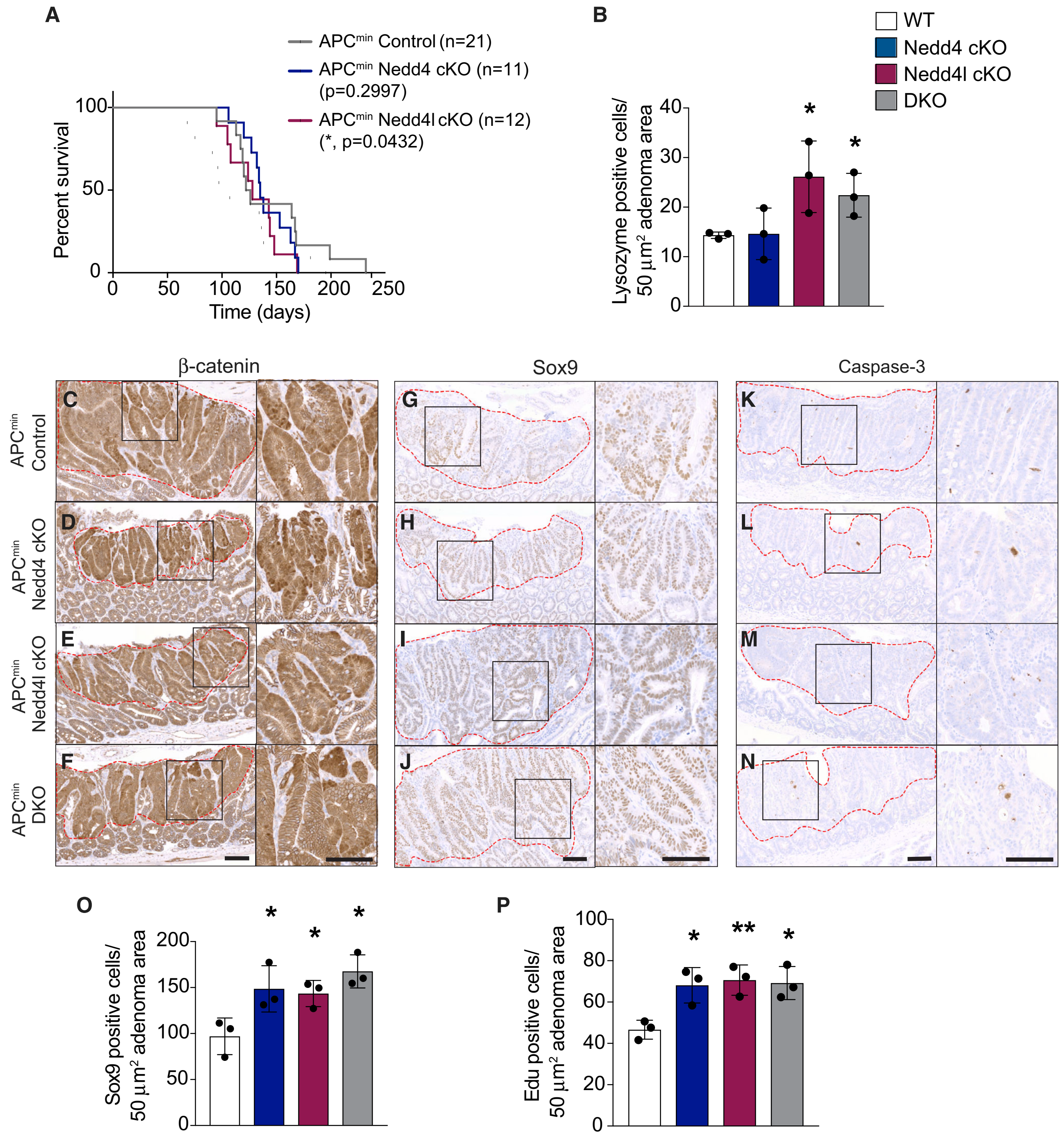

Figure EV3. Loss of Nedd4 and/or Nedd4l increases Wnt activation in Apc^{min} adenomas.

A Kaplan–Meier survival analysis of Apc^{min} control, Apc^{min} Nedd4 cKO and Apc^{min} Nedd4l cKO mice. *P*-values were determined using the Mantel–Cox test.

B Quantitation of lysozyme-positive cells in adenomas from the indicated mice. Each dot represents the average of at least 5 adenomas (with similar size and grade of dysplasia) per animal. Data are mean ± standard error. n = 3 per group.

C–N Immunostaining of adenoma tissues from Apc^{min} control (C, G, K), Apc^{min} Nedd4 cKO (D, H, L), Apc^{min} Nedd4l cKO (E, L, M) and DKO (F, J, N) mice using the indicated antibodies. Scale bars, 100 μm.

O–P Quantitation of Sox9-positive (O) and Edu-positive proliferating (P) cells per a defined area in the adenoma. Each dot represents the average of at least 5 adenomas (with similar size and grade of dysplasia) per animal. Data are mean ± standard error. n = 3 per group.

Data information: *P*-values were determined using the unpaired two-sided *t*-test (**P* < 0.05; ***P* < 0.01).

© 2019 The Authors

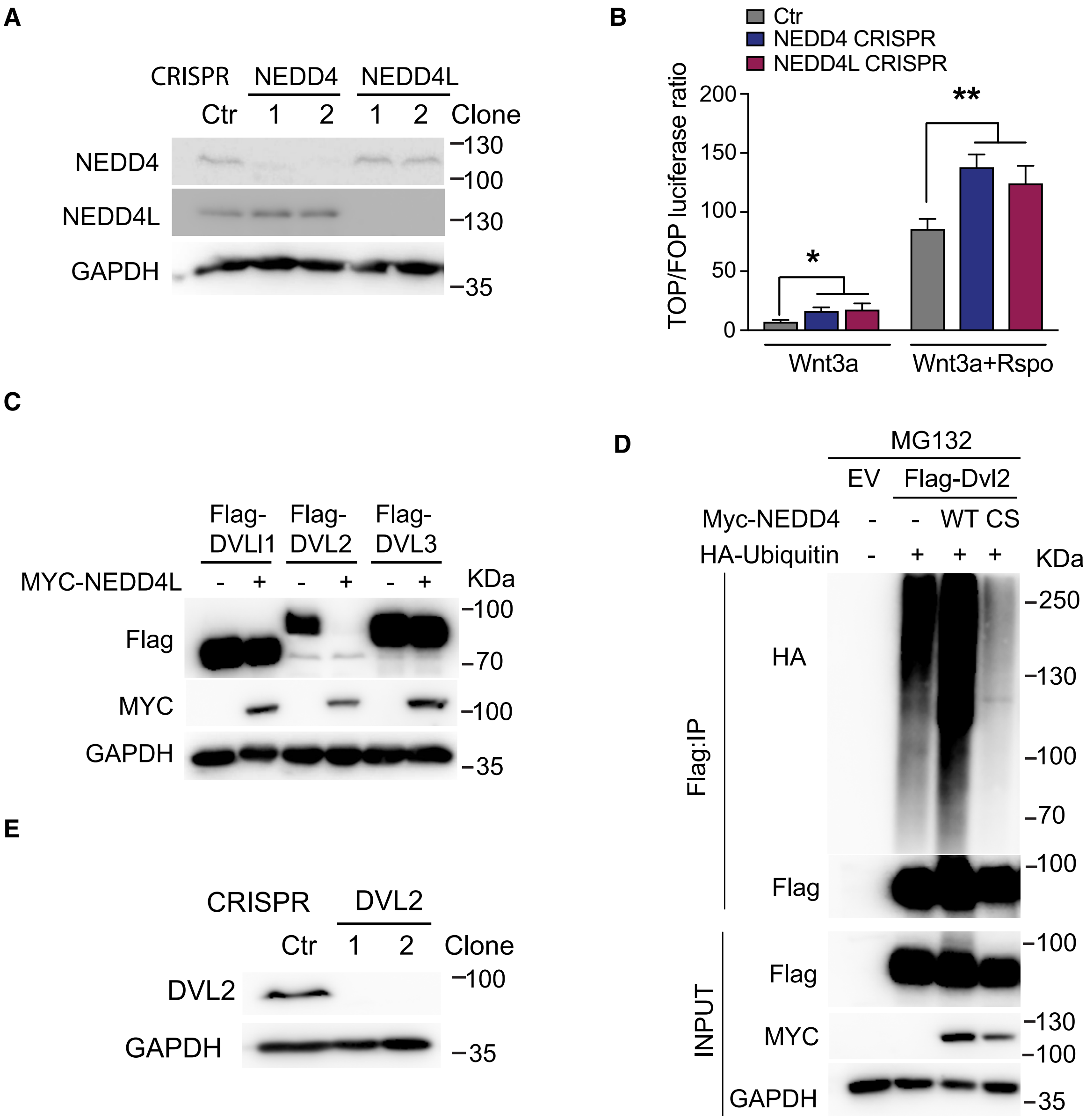

Figure EV4. NEDD4 and NEDD4L target DVL2 for degradation.

A Cell lysates of HEK293T control (Ctr) and the indicated CRISPR clones were analysed by Western blotting using the indicated antibodies.

B Relative TOPFlash reporter activities of HEK293T cells with the indicated CRISPR targeting. When indicated, cells were treated with Wnt3a-conditioned media or Wnt3a supplemented with RSPO. Data represent mean ± standard error of at least three independent experiments. *P*-values were determined using the unpaired two-sided *t*-test (*$P < 0.05$; **$P < 0.01$).

C Western blot analysis of HEK293T cells transfected with Flag-DVL1, Flag-DVL2 or Flag-DVL3 with or without MYC-NEDD4L using the indicated antibodies.

D HEK293T cells were transfected with constructs expressing Flag-DVL2, MYC-NEDD4-WT, Myc-NEDD4-CS mutant, HA-Ubiquitin or empty vector (EV), as indicated. Cells were treated with MG132, followed by anti-Flag IP and immunoblotting using the indicated antibodies.

E Cell lysates of HEK293T control (Ctr) and the indicated CRISPR clones were analysed by Western blotting using the indicated antibodies.

 © 2019 The Authors

Figure EV5. NEDD4 and NEDD4L selectively degrade Lgr4 and Lgr5 but not FZD receptors of Wnt signalling pathway.

A HEK293T cells were transfected with empty vector (EV), LGR4-Flag, MYC-NEDD4 WT or C854S (CS) mutant, MYC-NEDD4L wild-type (WT) or C962A (CA) mutant, followed by Western blot analysis of the indicated antibodies.

B, C HEK293T cells were transfected with V5-FZD4 (B) or V5-FZD5 (C) with or without MYC-NEDD4 or MYC-NEDD4L or empty vector (EV) as control. Lysates were subjected to Western blotting using the indicated antibodies.

D Subcellular localisation of SNAP-FZD5 in HEK293T cells co-expressed with the indicated plasmids. Surface SNAP-FZD5 was labelled with SNAP Alexa-488 for 10 min. Scale bars, 10 μm.

E Quantitation of fluorescent intensity in total and surface LGR5 and FZD5 with the indicated transfections of NEDD4 and NEDD4L. Data are presented as percentage of fluorescence intensity compared to the EV control in triplicate for LGR5 and duplicate for FZD5. Error bars represent ± SEM. *P*-values were determined using the unpaired two-sided *t*-test (*$P < 0.05$; ***$P < 0.001$).

F HEK293T cells WT, NEDD4 CRISPR or NEDD4L CRISPR mutants were transfected with LGR5-Flag. Twenty-four hours later, cells were treated with cycloheximide (Chx) (50 μg/μl) and were collected at different time points as indicated. Cell lysates were subjected to Western blot analysis using the indicated antibodies. Quantitation of the blots is shown at the bottom. Data represent mean ± standard error of at least three independent experiments. *P*-values were determined using the unpaired two-sided *t*-test compared between Nedd4 and WT (indicated by *) or Nedd4l and WT (indicated by #) at the same time point *$P < 0.05$; $^{\#\#}P < 0.01$).

G, H HEK293T cells were transfected with constructs expressing LGR5-Flag, HA-Ubiquitin, EV or the indicated NEDD4 (G) or NEDD4L (H) plasmids. Cells were treated with Bafilomycin A1 followed by anti-Flag IP and Western blot analysis using the indicated antibodies.

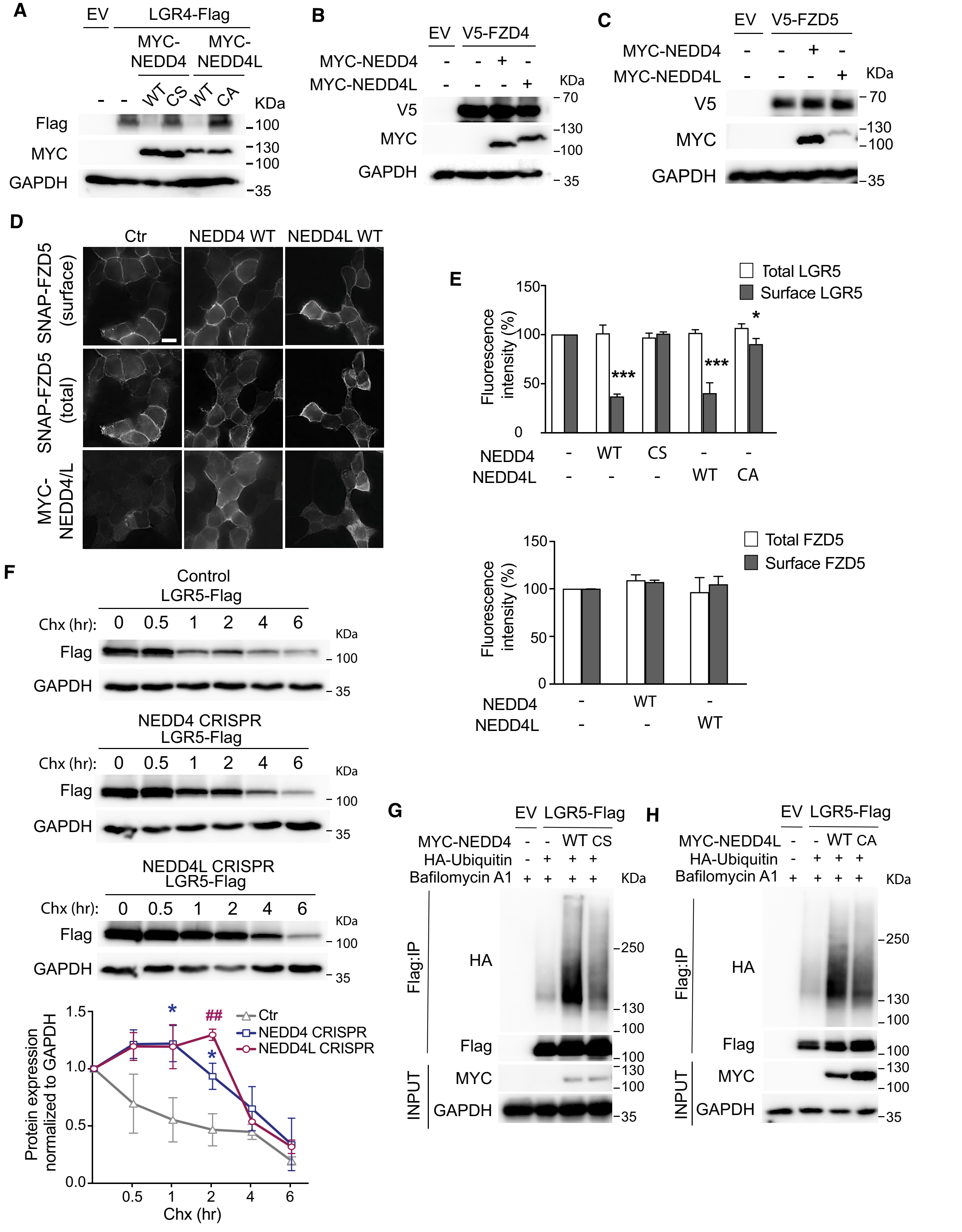

Figure EV5.

© 2019 The Authors
